# In Vitro Inhibition of Colorectal Cancer Gene Targets by *Withania somnifera* L. Methanolic Extracts: A Focus on Specific Genome Regulation

**DOI:** 10.3390/nu16081140

**Published:** 2024-04-12

**Authors:** John M. Macharia, Daniel O. Pande, Afshin Zand, Ferenc Budán, Zsolt Káposztás, Orsolya Kövesdi, Tímea Varjas, Bence L. Raposa

**Affiliations:** 1 Doctoral School of Health Sciences, Faculty of Health Sciences, University of Pécs, Vörösmarty Mihály Str. 4, 7621 Pécs, Hungary; 2 Department of Biological Sciences and Biomedical Science & Technology, School of Science and Applied Technology, Laikipia University, Nyahururu P.O. Box 1100-20300, Kenya; 3 Department of Public Health Medicine, Medical School, University of Pécs, 7621 Pécs, Hungary; 4 Institute of Physiology, Medical School, University of Pécs, 7621 Pécs, Hungary; budan.ferenc@pte.hu; 5 Faculty of Health Sciences, University of Pécs, 7621 Pécs, Hungary; 6 Institute of Basics of Health Sciences, Midwifery and Health Visiting, Faculty of Health Sciences, University of Pécs, Vörösmarty Mihály Str. 4, 7621 Pécs, Hungary

**Keywords:** *Withania somnifera* L., gene expression, colorectal cancer, carcinogenesis, phytotherapeutic inhibitors, in vitro application

## Abstract

An approach that shows promise for quickening the evolution of innovative anticancer drugs is the assessment of natural biomass sources. Our study sought to assess the effect of *W. somnifera* L. (WS) methanolic root and stem extracts on the expression of five targeted genes (cyclooxygenase-2, caspase-9, 5-Lipoxygenase, B-cell lymphoma-extra-large, and B-cell lymphoma 2) in colon cancer cell lines (Caco-2 cell lines). Plant extracts were prepared for bioassay by dissolving them in dimethyl sulfoxide. Caco-2 cell lines were exposed to various concentrations of plant extracts, followed by RNA extraction for analysis. By explicitly relating phytoconstituents of WS to the dose-dependent overexpression of caspase-9 genes and the inhibition of cyclooxygenase-2, 5-Lipoxygenase, B-cell lymphoma-extra-large, and B-cell lymphoma 2 genes, our novel findings characterize WS as a promising natural inhibitor of colorectal cancer (CRC) growth. Nonetheless, we recommend additional in vitro research to verify the current findings. With significant clinical benefits hypothesized, we offer WS methanolic root and stem extracts as potential organic antagonists for colorectal carcinogenesis and suggest further in vivo and clinical investigations, following successful in vitro trials. We recommend more investigation into the specific phytoconstituents in WS that contribute to the regulatory mechanisms that inhibit the growth of colon cancer cells.

## 1. Introduction

### 1.1. The Epidemiological Burden of Colorectal Cancer and Related Risk Indicators

Colon cancer is the fourth most common cancer globally, with rectum cancer ranking eighth in terms of incidence, according to GLOBOCAN 2018 data. When taken together, CRCs account for 11% of all cancer diagnoses worldwide, making them the third most common type of cancer diagnosed [1]. In developed economies, CRC currently ranks third in terms of cancer-related mortality [2]. In developing nations, cancer claims the lives of almost 70% of individuals [3], and this phenomenon is attributable to poorly managed, weakened, and constrained healthcare systems. Out of 191 countries worldwide, 10 have CRC as the most common cancer diagnosed in men; no country has CRC as the most common cancer diagnosed in women. Age-standardized (world) incidence rates per 100,000 of CRC in both sexes are 19.7, 23.6, and 16.3, respectively, for men and women. In high-HDI (human development index) countries, the age-standardized incidence rate for men is 30.1/100,000, while in low-HDI countries, it is 8.4. For women, the corresponding statistics are 20.9 and 5.9 [1,4]. The increased incidence of CRC is associated with high-fat diets, a low intake of whole grains, fruits, and vegetables, gender, age, race, and family history. It has also been determined that exposure to heavy metals like lead and infectious organisms poses risks for CRC [5]. Enhanced comprehension of the onset and progression pattern of colorectal cancer (CRC), genetic and environmental risk factors, and the evolutionary process of the disease can enable researchers and healthcare providers to mitigate the effects of this life-threatening cancer [1,6].

The burden of colorectal cancer (CRC) is largely attributed to industrialization and westernization. There is a notable shift in this global burden towards countries of lower economies as they continually become Westernized due to trade, tourism, professionalism, and other exchange programs across different continents. Overall, there are up to eight-fold differences in CRC incidence between countries depending on the geographical region. Incidence rates typically increase steadily with rising HDI in nations that are experiencing significant developmental transitions, indicating that there is a correlation [4].

### 1.2. An Overview of Targeted Genes in the Present Study

This study evaluated the expression of five genes implicated as significant targets for cancer management, namely, *COX-2*, *CASP9*, *Bcl2*, *Bcl-xL*, and *5LOX*. A significant protagonist in the control of apoptosis is *CASP9*, which signals the beginning of the mitochondrial caspase cascade. The caspase proteins are part of the chain reaction that is initiated by commands that support apoptosis and leads to the dissociation of many peptides and the fragmentation of cells. Comprehending caspase programming is essential for precisely regulating apoptosis in therapeutic settings [7,8]. Apoptosis is a crucial physiological process that involves the purposeful death of cells in a range of biological mechanisms [9]. There is a claim that impeding natural apoptosis increases the risk of malignancy [10,11]. In contrast, there is evidence that a higher frequency of colorectal adenoma is strongly associated with a lower rate of apoptosis [12].

Arachidonic acid is converted into inflammatory prostaglandins by the rate-limiting enzymes cyclooxygenase 1 and 2 (*COX-1* and *COX-2*). Cancer risk is increased by persistent inflammation [13]. When there are inflammatory disorders, *COX-2* is substantially stimulated. It is believed that *COX-2* selective inhibitors have negligible or no gastrointestinal adverse effects while having the comparable anti-inflammatory properties and antipyretic, and while considering the advantages of analgesia as a broad-spectrum antagonist NSAIDs [13]. Throughout the body, enzymes are essential in the organism’s metabolic processes, since they are engaged in the generation of lipid prostaglandins [14].

It is necessary to comprehend each member of the Bcl2 family’s propensity and concentration to comprehend the primary reactions that take place between them. The dominating interactions that dictate whether or not mitochondrial outer membrane permeabilization (MOMP) occurs are determined by these characteristics [15]. *Bcl-xL* is present in the cytoplasm, extra-nuclear membranes like the mitochondrion, and the nuclear envelope, whereas *Bcl2* is found in the mitochondrion, endoplasmic reticulum (ER), and the nuclear envelope [16]. The precise methods of action of *Bcl2* and *Bcl-xL* are complex, and numerous interactions with other proteins have been postulated. It is unknown how important a given interaction is for the final phenotype at the cellular level [17]. It has been demonstrated that prexasertib management and siRNA-mediated *Bcl-xL* downregulation greatly boost apoptosis. Moreover, it has been demonstrated that prexasertib plus navitoclax exhibits a potent antitumor impact and suppresses *Bcl-xL* to cause apoptosis in malignant cells [9]. 

There is a wide range of lipoxygenases (LOXs) in fungi, bacteria, plants, and animals. These are iron-containing, non-heme enzymes. The Ca^2+^ and ATP-dependent enzyme *5LOX* catalyzes the first two stages in the synthesis of the peptide-LTs and the chemoattractant factor LTB4. Granulocytes, mast cells, monocytes/macrophages, and B lymphocytes are myeloid cells that express the *5LOX* protein genome [18,19].

### 1.3. Phytotherapeutic Approaches to Treatment

The development of cancer drug resistance and its related side effects are closely linked to synthetic treatments for CRC. This has prompted research into natural alternatives as potential strategic options. But even with this powerful natural phytotherapeutic approach, research and exploration into the useful medicinal plants now in use is lacking [6,20,21]. The progress in pharmacotherapeutics is hindered by a lack of understanding of the current plant metabolites, their biological roles, and the processes involved in their extraction. Plant-derived phytoconstituents have been shown to exhibit potent therapeutic effects in the treatment of a wide range of infectious diseases, making them less likely than synthetic drugs to cause a variety of side effects [22]. Emerging anti-colorectal cancer therapies have largely come from organic substances derived from plants; they directly or indirectly contribute to almost half of all anticancer medicines currently in use. We have previously investigated and reported on the safety and cytotoxicity characteristics of WS, which informed the need for further exploration [20]. The plant is a significant but little-studied plant species, natively occurring in Kenya, Africa. 

### 1.4. The Botanical Description and Global Distribution of W. somnifera (L.) 

According to the biological classification system, *Withania somnifera* (L.) is a species of plant that is a member of the kingdom Plantae (plants), the sub-kingdom Tracheophytes (vascular plants), division Angiospermae, class Eudicots, clade Asterids, order Solanales, family Solanaceae, sub-family Solanoideae, tribe Physale-ae, genus *Withania*, and species *somnifera* [23,24,25]. *W. somnifera* L. is a small shrub that grows abundantly in the subtropical regions and is frequently referred to as “Ashwagandha” in Hindi and Sanskrit. It grows in the dry tropical regions of Afghanistan, Pakistan, South and East Africa, Spain, Sri Lanka, Sudan, China, Congo, India, Egypt, Israel, Jordan, Madagascar, Morocco, Nepal, and the Canary Islands [26]. The leaves and roots of this plant are used in Ayurveda, which is a traditional Indian medicine [27]. Ayurveda is a well-respected traditional medical system with a long history. This approach uses a variety of natural chemical substances in different ways to meet its therapeutic objectives [28]. Thousands of herbs, including *Withania somnifera* L., are beneficial in avoiding illnesses and preserving health according to the Ayurvedic system [20]. 

Our study sought to assess the effect of *W. somnifera* L. (WS) methanolic root and stem extracts on the expression of five targeted genes (cyclooxygenase-2, caspase-9, 5-Lipoxygenase, B-cell lymphoma-extra-large, and B-cell lymphoma 2) in colon cancer cell lines (Caco-2 cell lines).

## 2. Methodology

### 2.1. Procurement of Caco-2 Cell Lines

The Department of Biochemistry and Medical Chemistry at the University of Pecs offered Caco-2 cell lines directly to our laboratory (the Department of Public Health) following procurement from the ATCC (American Type Culture Collection). Caco-2 has potential use in toxicity and cancer studies and are excellent hosts for transfection. The cancer cell lines were preserved in compliance with the manufacturer’s instructions [29].

### 2.2. Plant Organ Acquisition

Organs of WS were obtained from the Perkerra irrigation scheme, Baringo South Sub-County in Baringo County (0°28′42″ N · 36°1′38″ E · 0.4786 Latitude, and 36.0274 Longitude). The reclamation scheme lies near Marigat Township, around a hundred kilometers north of Nakuru City. It derives its name from River Perkerra, which is the only perennial natural river in the area and a source of water for irrigation [30]. The organ samples were then transported to Egerton University, Kenya, for further processing. A taxonomist performed an identification of the plant species, and a voucher specimen was collected and deposited in the Egerton University’s herbarium.

### 2.3. Extraction of WS Extracts Using Methanol and Acquisition of the Plant Organs

After being shade-dried, the chosen stem and root organs were finely powdered. The solvent used for serial exhaustive extraction (SEE) was methanol. To extract phytoconstituents, 1000 g of WS organ of the plant was immersed in a glass container and extracted for three days with continuous shaking using ethyl acetate. Upon undergoing filtration through Whatman filter paper with particle sizes ranging from 4 to 1 in diameter, crude solvent extracts were dechlorophyllated. To ensure that all soluble components were maximally extracted, this process was repeated three times [31]. For maximum cell penetration, a recommended minimum volume of 70% methanol (MeOH) was utilized. Essentially, 70–80% MeOH is the most widely utilized solvent because it has strong cell content penetration and is therefore suitable for extracting all primary and secondary metabolites [32,33,34,35,36]. The solvent was evaporated in order to concentrate the extract. A rotor evaporator (Marshall Scientific LLC., Hampton, NH, USA) operating at lower pressure and temperatures between 40 and 50 °C was used to remove the solvent. A freezer dryer (Azbil Telstar, SLU, Barcelona, Spain) was used to lyophilize the aqueous extract. Tightly stoppered vials were used to keep the dry, solvent-free metabolites before use [37]. The desiccator was kept at 4 °C in a refrigerator.

### 2.4. Reconstitution of Plant Extracts

Dimethyl sulfoxide (DMSO) was applied in dissolving plant extracts for bioassays [38]. It served as both a suspending medium and an inert diluent for crude plant extracts that were insoluble in water. Using double-distilled phosphate-buffered saline (ddPBS) as the diluent solvent and 0.5% DMSO as the dissolving solvent, a 30 mg/mL stock solution was created. The final concentrations of 2 mg/mL, 1 mg/mL, and 0.5 mg/mL for the treatment of Caco-2 cell lines were then made using the stock solution.

### 2.5. Passaging Cancer Cell Lines (Caco-2)

The Caco-2 cell-containing culture T-flask (75 cm^2^ or 175 cm^2^) was carefully placed inside a lamina hood and kept sterile. The used media was drawn out once the culture T-flask was opened. PBS was used to wash it twice. After covering, PBS-EDTA was applied and left for a short while. It was then gently and carefully pipetted. Two milliliters of trypsin was used to dissociate and detach Caco-2 cells from the surface and clumps, respectively. The surface was treated with trypsin by gently gliding the flask over it from side to side. The flask was placed inside the thermostat and left for five minutes. Five minutes later, the flask was removed, and Caco-2 medium was judiciously poured once there was sufficient apparent separation. The adherent Caco-2 cell type has a tendency to stick to surfaces. After being pipetted into a tube, the entire contents of the flask were centrifuged for five minutes at 125 rpm. The Caco-2 cells were then left at the tube’s bottom, and the supernatant was pipetted out. The cells were gently shaken with a pipette by pipetting up and down after adding fresh media to the tube. Fresh culture T-flasks were filled with medium, the suspension was separated, and the growth thermostat was placed within. Growth conditions were maintained at 95% air, 5% CO_2_, and 37 °C [39]. To allow for treatment, confluence was observed until 70–80%.

### 2.6. Treatment of Cancer Cell Lines with WS Extracts

An amount of 200 μL of extract solutions at varying concentrations levels (2 mg/mL, 1 mg/mL, and 0.5 mg/mL) was applied to passaged Caco-2 cell lines in fresh media. Following treatment, the cells were cultured for 36 h at 37 °C. A light microscope was used to examine the condition of the cells after incubation. The typical doubling time of cancer cell lines, which is between 36 and 48 h, was used to establish the exposure length of interventions (36 h). The cells received doses at different concentration levels, starting with 0.5 mg/mL to evaluate the cells’ response to different doses and the time taken for significant reactions to occur. This allowed for the precise detection and evaluation of the regulatory properties that increased with dose level of concentration. Growth and possible physiological responses were noted at 12 h intervals.

### 2.7. RNA Isolation

Following the removal of the medium from the cell cultures, it underwent two PBS washes and a trypsin-EDTA treatment. Following centrifugation, the cell suspension was pipetted into a 4 cm^3^ centrifuge tube. After adding 1 cm^3^ of ExtraZol Tri-reagent solution, it was left to incubate at room temperature for 5 min. Chloroform (0.2 cm^3^) was added. The sample was centrifuged at 12,000× *g* for 10 min at 2–8 °C after being incubated for 2–3 min. A sanitized tube was used to hold the aqueous phase. Isopropyl alcohol (0.2 cm^3^) was added. The material was centrifuged once more at 12,000× *g* for 10 min at 2–8 °C following a 10 min incubation period. Then, 1 cm^3^ of 75% alcohol was used to wash the RNA pellet after the supernatant was removed. It was vortexed, then centrifuged for 5 min at 2–8 °C at 7500× *g*. Following the removal of the supernatant, the particulate was dried. Next, 50–100 µL of DEPC water that was devoid of RN-ase was used to dissolve it. Subsequently to vortexing, the sample was incubated at 55 °C for 10 min. Up until it was used, the extracted RNA was preserved at −80 °C.

### 2.8. RNA Concentration and Purity Assessment Using UV Spectroscopy

Ultraviolet (UV) spectroscopy was employed to evaluate both the concentration and purity of RNA. To maximize the effectiveness of this procedure, RNA samples were first treated with RNAse-free DNAse to eliminate contaminating DNA. Throughout the procedure, extra impurities including leftover proteins and phenol that can affect absorbance measurements were carefully eliminated. At 260 and 280 nm, the absorbance of a diluted RNA sample was measured. The Beer–Lambert equation, which states that absorbance will change linearly with concentration, was used to quantify the concentration of nucleic acid. When RNA purity was measured using the A260/A280 ratio, a ratio of 1.8 2.1 indicated highly pure RNA. 

### 2.9. Equipment and Procedure for a Quantitative Real-Time PCR (SYBR Green Protocol)

Accurate, repeatable, and sensitive nucleic acid quantification is made possible by qRT-PCR. Observing the guidelines provided by the manufacturer, a Roche LightCycler 480 qPCR (Thermo Fisher Scientific, Rockford, IL, USA) platform with 96-well plates was utilized for one-step PCR, comprising proliferation and reverse transcription. The One-Step Detect SyGreen Lo-ROX one-step RT-PCR kit (Nucleotest Bio Ltd., Budapest, Hungary) was used for the procedure. At the end of each of the forty-five cycles (95 °C-5 s, 56 °C-15 s, and 72 °C-5 s), a fluorescence output was acquired in the thermal program, which was set up as follows: 42 °C for 5 min of incubation, followed by 95 °C for 3 min. Melting curve analysis (95 °C-5 s, 65 °C-60 s, 97 °C∞) was performed after each run to verify the specificity of the amplification. The following was the reaction mix: upon adding 5 μL of mRNA template fortified with sterilized double-distilled water, 10 μL of Master Mix, 0.4 μL of RT Mix, 0.4 μL of dUTP, and 0.4 μL of primers, an aggregate amount equal to 20 μL was attained. The primers were developed by Integrated DNA Technologies (Bio-Sciences Ltd., Budapest, Hungary), and the sequences were constructed with the Primer Express™ Software v3.0.1 package as shown in Table 1, below.

### 2.10. Analysis of Quantitative Real-Time PCR

Using qRT-PCR high-throughput detection and quantification matrices of target DNA sequences, the relative gene expressions of *CASP9*, *Bcl-xL*, *5-LOX*, *Bcl2*, and *COX-2* targeted genes were determined. For internal control, the house-keeping gene used in our experimental study was *HPRT1.* The cross point between the threshold value and the amplification curve was shown by the Cp values used to express the PCR results. The proportional variations of the desired genes from the reference sample were calculated by applying the 2-Cp (Livak strategy) and the Cp values [40].

### 2.11. Data Analysis

IBM SPSS Version 26.0.3 (IBM Corp., 2019, Armonk, NY, USA) and MS Excel 2013 (Microsoft Corp., 2013, Redmond, WA, USA) were employed in the quantitative evaluation’s computation. Following a normality examination of the data using the Kolmogorov–Smirnov test and the analysis of variance (ANOVA), the mean values of the pertinent variables were compared. The outcome was regarded as significant if it was *p* ≤ 0.05 within the 95% confidence interval.

## 3. Results

### 3.1. Responses of COX-2 following Administration of Methanolic Stem and Root Extracts at Progressive Dose Concentrations

**Figure 1:**
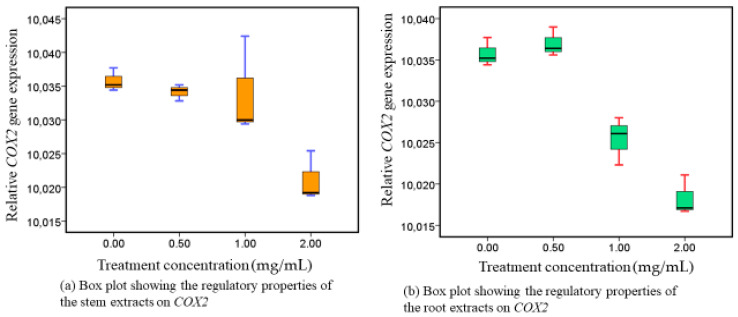
*COX-2* responses after exposure to stem and root extracts.

Methanolic extracts of WS were administered to Caco-2 cell lines at progressively higher doses of 0.00 mg/mL, 0.50 mg/mL, 1.00 mg/mL, and 2.00 mg/mL. In both extracts, *COX-2* transcripts were gradually inhibited in a dose-responsive way (Figure 1). Additionally, a statistically significant distinction was identified in the inhibitory responses from stem extracts (*p* = 0.010) and root extracts (*p* = 0.001), as shown in Table S1 (Supplementary Materials).

### 3.2. CASP9 Reactions following Exposure to Intervention Therapy

**Figure 2:**
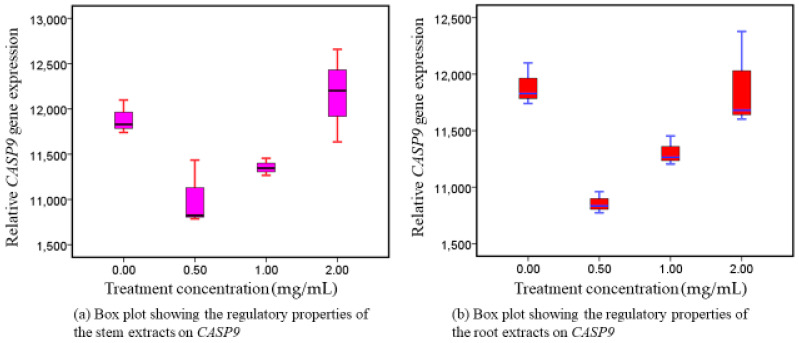
CASP9 responses following exposure to intervention therapy (stem and root extracts).

Following exposure of Caco-2 to intervention therapy (root extracts and stem extracts) of WS, there was increased (upregulation) expression of *CASP9* genes in both extracts, in a fashion dependent on dose concentration, (Figure 2). The two extracts differed noticeably from one another: *p =* 0.002 in root extracts and *p* = 0.011 in stem extracts (Table S1). The highest upregulatory activity was observed at higher concentrations. *CASP9*, which initiates the mitochondrial caspase mechanism, is an indispensable intermediary in the control of apoptosis.

### 3.3. Responses of Bcl-xL following Administration of Methanolic Stem and Root Extracts at Progressive Dose Concentrations

**Figure 3:**
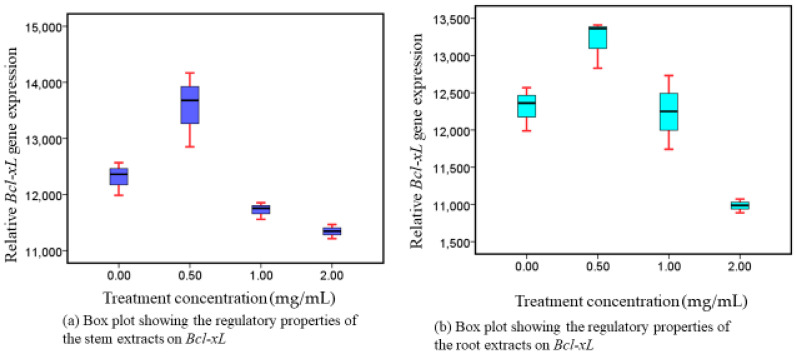
*Bcl-xL* expressions following exposure to intervention.

Following the exposure of Caco-2 cell lines to intervention, the expression of *Bcl-xL* genes was downregulated in a dosage-dependent way, in both extracts (Figure 3). There was a significant difference in their downregulatory potential (*p* = 0.001, roots and *p* = 0.001, stems) noticed in both extracts. Restricted *Bcl-xL* activation corresponds to enhanced apoptotic implications, which are essential for preventing the growth of malignant cells.

### 3.4. Bcl2 Expressions following Exposure to Intervention

**Figure 4:**
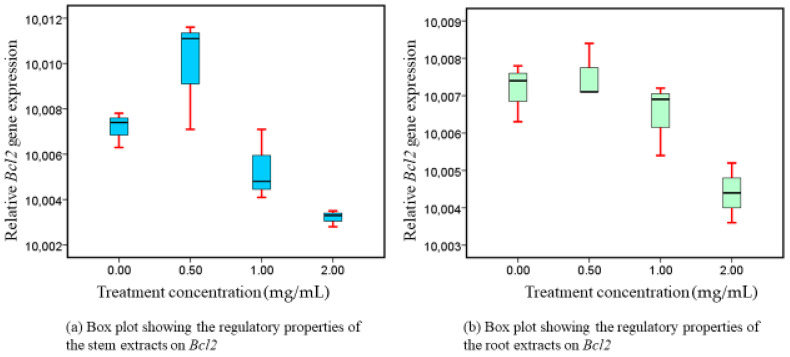
*Bcl2* expressions following exposure to intervention.

There was a dose-related downregulation of *Bcl2* gene activity, in both extracts (Figure 4). Their respective downregulatory effects differed significantly (*p* = 0.007, roots and *p* = 0.004, stems) recorded in both extracts. A variety of apoptosis-stimulating active metabolites found in WS are responsible for the distinctive downregulatory properties observed in *Bcl2*, as reported in our study.

### 3.5. 5-LOX Responses following Administration of Methanolic Stem and Root Extracts at Progressive Dose Concentrations

**Figure 5:**
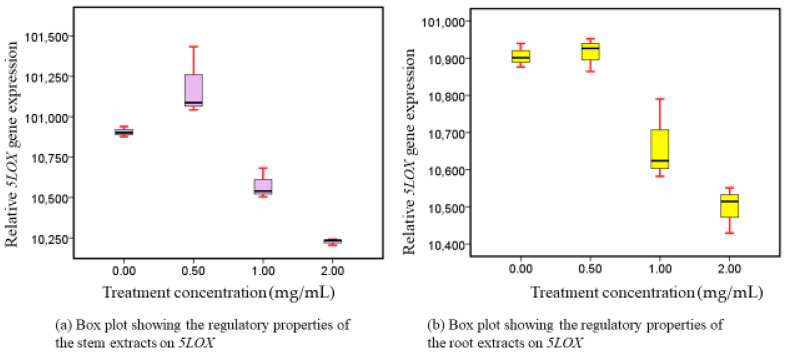
*5LOX* following exposure to stem and root-extracts intervention.

The activity of 5-LOX genes was suppressed in both extracts when Caco-2 cell lines were administered to them, in a mechanism that was dependent on the administered dose amount (Figure 5). In both extracts, there was also a noteworthy variation in their inhibitory characteristics (*p* = 0.001).

## 4. Discussion

### 4.1. Phytotherapeutic Effects of Both (Roots and Stem) Extracts on Cyclooxigenase-2 Modulation

While COX-1 modifies balance, COX-2 is heavily involved in inflammatory reactions [41]. Although COX-2 expression in the colon is modest, it can be impacted by lipopolysaccharides, growth hormones, necrosis-related factors, and cytokines in adverse circumstances. Higher concentrations of COX-2 are linked to the onset and progression of colorectal cancer [42]. The results from our experimental study demonstrated that upon exposure of Caco-2 cell lines on the extracts, *COX-2* genes were dose-dependently inhibited (Figure 1). The copious distribution of the biochemically functional metabolites that are already present in both roots and stems accounts for the considerable advantage in the inhibition of COX-2 demonstrated in our investigation. These metabolites include, among others, 27-*O*-glucopyranosylviscosalactone B, 4,16-dihydroxy-5 h, 6h-epoxyphysagulin D, diacetylwithaferin A, physagulin D (1-6)-h-D-gluco-pyranosyl-(1-4)-h-D-glucopyranoside, viscosalactone B, withaferin A, withanolide sulfoxide, and withanoside IV, each of which has been linked to inhibiting *COX-2* activity [43,44,45]. Notably, then, the roots and stems of WS may provide a viable substitute for conventional *COX-2* antagonists used in phytotherapy, which are known to have adverse reactions [5].

### 4.2. Phytotherapeutic Effects of Both Extracts on CASP9 Regulation

Our findings showed that exposure to root and stem extracts varied in the overexpression of *CASP9* in Caco-2 cells, suggesting that WS are appealing inducers of cell death responses. It was shown that *CASP9* transcripts progressively elevated in a dose-responsive way after Caco-2 cell lines were exposed to the interventions (Figure 2). The capacity to cause cells in the gastrointestinal tract to undergo apoptosis is one possible preventive chemotherapy tactic [11]. It has been proposed that suppressing spontaneous cell death increases the risk of cancer [10,11]. Comparably, a decreased rate of apoptosis is highly linked with a higher frequency of colorectal adenoma [12]. Therefore, studying the cell death cascade is a good way to treat CRC. The activity level of the apoptosis-associated markers (*CASP9* and *CASP10*) may help assess the prognosis for patients with stage II colorectal cancer. The downregulation of *CASP9* and *CASP10* increases the lifetime of abnormal mucosa cells [46,47]. Because of this, these cells may experience additional gene alterations, which could ultimately result in the development of malignant cells. The regulation of nuclear factor-kappa B (NF-kB) activity has been proposed as a mechanism by which withanolides exert their action. This is because NF-kB controls the activity of many genes involved in cell division, cancer metastasis, and inflammatory reactions. This could potentially explain why withanolides can promote apoptosis while limiting invasion, as it implies that they inhibit NF-kB activity and NF-kB-regulated gene expression [48]. Withanolide D has been shown to decrease the levels of anti-apoptotic genes (TERT, Bcl-2, and Puma) in a study conducted in a leukemic murine mice model [49]. At an IC50 of 92 g/mL, mitochondria-mediated apoptosis in triple-negative breast cancer cells (MDA-MB-231) could be induced by dysregulated Bax/Bcl2 expression and simultaneous disruption of mitochondrial membrane potential (ΔΨm), a novel fraction of proteins isolated from WS-roots, under conditions of high levels of reactive oxygen species (ROS).

*CASP3* activation, G2/M cell cycle arrest, and nuclear lamina protein disintegration were also described [50]. Additionally, pro-apoptotic and tumor-promoting proteins in the signaling cascade were altered by the crude water extract (0.5%) of WS, which contributed to the inhibition of tumor growth [49]. Utilizing Withaferin A exposure has been reported to increase the number of late apoptotic cells and the aggregation of cells in the cell cycle’s subG1 arrest. It has been connected to the cleavage of *CASP-3* and PARP, which induces apoptosis [44,51,52,53]. In light of the aforementioned findings, investigating the dose-related apoptotic mechanisms of related genes, as demonstrated in our unique research and conclusions, is a viable pharmacotherapeutic therapy approach for the management of colorectal cancer. However, additional in vitro research findings are necessary to verify the current findings, prior to transitioning to in vivo experimental trials.

### 4.3. Phytotherapeutic Effects of Both Extracts on Bcl-xL and Bcl2 Regulation

Pro- and anti-apoptotic components of the Bcl-2 protein group are among the best-defined groups of proteins associated with the regulation of cell death. Components of this family with anti-apoptotic properties, such *Bcl2* and *Bcl-xL,* block apoptosis by either binding proforms of the death-inducing cysteine proteases, or by stopping the elimination of mitochondrial apoptogenic substances, like cytochrome c and AIF (apoptosis-inducing factor), from their bodies. Cytochrome c and AIF effectively activate caspases as they enter the cytoplasm. Caspases subsequently degrade several cellular proteins, inducing apoptosis [9]. Our research showed that, in a dose-related way, stem preparations from WS dramatically reduced the level of expression of *Bcl-xL* and *Bcl2*. Comparable modulatory responses were distinctively expressed by both genes. That being said, the reason for their comparable activity is that they share a common anti-apoptotic genetic group [15]. Reduced *Bcl-xL* and *Bcl2* expression is associated with enhanced apoptotic effects, which are essential for inhibiting the growth of malignant cells. Our findings suggest that *Bcl-xL* and *Bcl2* promote programmed cell death [54] and may be appropriate candidates for CRC intervention. The treatment extracts presented in our investigation exhibited a distinctive suppression of *Bcl-xL* and *Bcl2*, which we attribute to the presence of a variety of apoptosis-stimulating functioning metabolites that were previously addressed as being present in WS [44]. It has been reported that withaferin A increases apoptosis through *PARP* and *CASP3* degradation and decreases levels of anti-apoptotic proteins such as *Bcl-xL* and *Bcl2* [44,51,52,53]. 

According to our empirical studies, *Bcl-2* and *Bcl-xL* inhibition amplifies their reduced activity, which in turn leads to a reduction in the growth of Caco-2. Pharmacologic modulation of proteins from the Bcl-2 family will be limited until a more precise knowledge of how these molecules regulate cell apoptosis is obtained. Despite our effective in vitro utilization, our investigation highlights the need for more studies on Bcl-2 family connections and their pharmacological modification utilizing the commercially available WS plant-based constituents in vivo. For the very first time, we report that WS methanolic root and stem extracts potency is essential in regulating *Bcl2* and *Bcl-xL.* In addition, their corresponding intracellular signaling pathways inhibit CRC growth. The utilization of methanol for effective extraction of *Bcl-xL* and *Bcl2* modulatory phytoconstituents is highly recommended from the results of our findings.

### 4.4. Phytotherapeutic Effects of Both Extracts on 5-LOX Regulation

It appears that disrupting these channels may be useful in delaying the advancement of CRC and other malignancies, as some LOXs create intermediates (metabolites) in the arachidonic acid route that appear to promote carcinogenesis [55,56,57]. 5-LOX and its products Leukotriene (LT)-B4 and 5(S)-hydroxy-6E,8Z,11Z and 14Z-eicosatetraenoic acid (5-S-HETE) are two of these LOXs and intermediates [56]. Thus, 5-LOX is a dioxygenase that converts arachidonic acid to 5-S-HETE, which is further converted to LTB4 by LTA4 hydrolase [58]. Our findings demonstrated that the level of activity of *5-LOX* was dramatically inhibited in a dose-related way by the utilized methanolic extracts (roots and stems). Therefore, it was concluded that methanol is a remarkable extraction solvent for obtaining the highest concentration of beneficial metabolites that can inhibit the expressive characteristics of 5-LOX. 

It is unclear what steps lead to the activity of the 5-LOX genome during neoplastic transformation; however, 5-LOX activity does seem to be periodically enhanced [56]. LOX inhibitors reduce the development of cancerous cells both in vivo and in vitro and cause death through mitochondrial pathways [57,58]. WS preparations with an IC50 value of 0.92 mg/mL and a 65% suppression capacity for 5-LOX have also been reported by other researchers [18]. The results of this study and additional studies suggest that 5-LOX communication cascade suppression and upregulation may be potential targets for both the prevention and the therapy of CRC. Thus, this study validates the use of WS natural 5-LOX inhibitory compounds for the therapeutic management of human colorectal cancer.

## 5. Conclusions

In this study, multiple dosages of *W. somnifera* L. methanolic extract were applied in vitro to Caco-2 cell lines. The stem and root extracts were empirically demonstrated to be of exceptional effectiveness in modifying the functional characteristics of cyclooxygenase-2, caspase-9, 5-Lipoxygenase, B-cell lymphoma-extra-large, and B-cell lymphoma 2. In addition, our results demonstrated that methanol is a viable extraction solvent of powerful metabolites that significantly modulates the expression of CRC associated genes. Therefore, utilizing WS as a potential colon cancer inhibitor with strong phytotherapeutic effects makes it plausible, due to the abundance of phytoconstituent biomolecules present in the plant. 

The regulatory effects observed in this study solidify WS’s status as a valuable plant of choice, with its stem and root extracts demonstrating substantial suppressive characteristics of CRC. This study is among the first to establish a direct correlation between the useful properties of WS preparations and specifically selected genomes, and possible medicinal applications for CRC management. However, more in vitro works are essentially required to corroborate the current conclusions. In this regard, we strongly suggest the utilization of WS animal models (in vivo) and later on in clinical applications, following additional and successful in vitro experimental trials. Further, we recommend the application of methanol as an excellent solvent for the extraction of bioactive metabolites in WS, to optimize regulatory benefits in the activities of cyclooxygenase-2, caspase-9, 5-Lipoxygenase, B-cell lymphoma-extra-large, and B-cell lymphoma 2. Lastly, we endorse further investigation into the particular phytochemicals linked to the regulatory mechanisms that prevent the development and advancement of CRC cells.

## Figures and Tables

**Table 1:** Forward and reverse primer sequences adopted and applied in our experimental study.

Primer ID	Forward Primer	Reverse Primer
Cyclooxygenase-2	CGGTGAAACTCTGGCTAGACAG	GCAAACCGTAGATGCTCAGGGA
5-Lipoxygenase	GGAGAACCTGTTCATCAACCGC	CAGGTCTTCCTGCCAGTGATTC
B-cell lymphoma 2	ATCGCCCTGTGGATGACTGAGT	GCCAGGAGAAATCAAACAGAGGC
B-cell lymphoma-extra-large	GCCACTTACCTGAATGACCACC	AACCAGCGGTTGAAGCGTTCCT
Caspase-9	GTTTGAGGACCTTCGACCAGCT	CAACGTACCAGGAGCCACTCTT
*HPRT1*	TGCTTCTCCTCAGCTTCA	CTCAGGAGGAGGAAGCC

## Data Availability

The datasets generated and/or analyzed during the current study are available from the corresponding author on reasonable request. The data are not publicly available due to its sensitive nature.

## Supplementary Materials

The following supporting information can be downloaded at: https://www.mdpi.com/article/10.3390/nu16081140/s1, Table S1. Results of the correlation between the content of methanolic extracts and the desired genes’ activity.
