# Supplementary material for: In Vitro Inhibition of Colorectal Cancer Gene Targets by Withania somnifera L. Methanolic Extracts: A Focus on Specific Genome Regulation
Source: Nutrients. 2024 Apr 12;16(8):1140. doi: 10.3390/nu16081140 (PMC11054881; doi:10.3390/nu16081140)
Supplement: Supplementary file 1 [file nutrients-16-01140-s001.zip › nutrients-2931313-supplementary.pdf]

Table S1. Results of the correlation between the content of methanolic extracts and the desired genes' activity

Dependent Variable	(I) Conc	(J) Conc	Mean Difference (I-J)	Std. Error	Sig.	95% Confidence Interval	
						Lower Bound	Upper Bound
COX2	.00	.50	.0016333	.0034642	.963	-.009460	.012727
		1.00	.0018333	.0034642	.950	-.009260	.012927
		2.00	.0146333*	.0034642	.012	.003540	.025727
	.50	.00	-.0016333	.0034642	.963	-.012727	.009460
		1.00	.0002000	.0034642	1.000	-.010894	.011294
		2.00	.0130000*	.0034642	.023	.001906	.024094
	1.00	.00	-.0018333	.0034642	.950	-.012927	.009260
		.50	-.0002000	.0034642	1.000	-.011294	.010894
		2.00	.0128000*	.0034642	.025	.001706	.023894
	2.00	.00	-.0146333*	.0034642	.012	-.025727	-.003540
		.50	-.0130000*	.0034642	.023	-.024094	-.001906
		1.00	-.0128000*	.0034642	.025	-.023894	-.001706
Lox5	.00	.50	-.2819333	.0969382	.076	-.592364	.028497
		1.00	.3304333*	.0969382	.037	.020003	.640864
		2.00	.6788333*	.0969382	.001	.368403	.989264
	.50	.00	.2819333	.0969382	.076	-.028497	.592364
		1.00	.6123667*	.0969382	.001	.301936	.922797
		2.00	.9607667*	.0969382	.000	.650336	1.271197
	1.00	.00	-.3304333*	.0969382	.037	-.640864	-.020003
		.50	-.6123667*	.0969382	.001	-.922797	-.301936
		2.00	.3484000*	.0969382	.029	.037970	.658830
	2.00	.00	-.6788333*	.0969382	.001	-.989264	-.368403
		.50	-.9607667*	.0969382	.000	-1.271197	-.650336
		1.00	-.3484000*	.0969382	.029	-.658830	-.037970
Bcl2	.00	.50	-.0027667	.0012437	.196	-.006749	.001216
		1.00	.0018333	.0012437	.493	-.002149	.005816
		2.00	.0039667	.0012437	.051	-.000016	.007949
	.50	.00	.0027667	.0012437	.196	-.001216	.006749
		1.00	.0046000*	.0012437	.025	.000617	.008583
		2.00	.0067333*	.0012437	.003	.002751	.010716
	1.00	.00	-.0018333	.0012437	.493	-.005816	.002149
		.50	-.0046000*	.0012437	.025	-.008583	-.000617
		2.00	.0021333	.0012437	.376	-.001849	.006116
	2.00	.00	-.0039667	.0012437	.051	-.007949	.000016
		.50	-.0067333*	.0012437	.003	-.010716	-.002751
		1.00	-.0021333	.0012437	.376	-.006116	.001849
BclXL	.00	.50	-1.2612333*	.3086234	.015	-2.249554	-.272913
		1.00	.5834667	.3086234	.304	-.404854	1.571787
		2.00	.9632000	.3086234	.056	-.025121	1.951521
	.50	.00	1.2612333*	.3086234	.015	.272913	2.249554
		1.00	1.8447000*	.3086234	.001	.856379	2.833021
		2.00	2.2244333*	.3086234	.000	1.236113	3.212754
	1.00	.00	-.5834667	.3086234	.304	-1.571787	.404854
		.50	-1.8447000*	.3086234	.001	-2.833021	-.856379
		2.00	.3797333	.3086234	.627	-.608587	1.368054

	2.00	.00	-.9632000	.3086234	.056	-1.951521	.025121
		.50	-2.2244333*	.3086234	.000	-3.212754	-1.236113
		1.00	-.3797333	.3086234	.627	-1.368054	.608587
CASP 9	.00	.50	.8753333*	.2703405	.048	.009608	1.741059
		1.00	.5337333	.2703405	.273	-.331992	1.399459
		2.00	-.2762667	.2703405	.742	-1.141992	.589459
	.50	.00	-.8753333*	.2703405	.048	-1.741059	-.009608
		1.00	-.3416000	.2703405	.608	-1.207325	.524125
		2.00	-1.1516000*	.2703405	.012	-2.017325	-.285875
	1.00	.00	-.5337333	.2703405	.273	-1.399459	.331992
		.50	.3416000	.2703405	.608	-.524125	1.207325
		2.00	-.8100000	.2703405	.067	-1.675725	.055725
	2.00	.00	.2762667	.2703405	.742	-.589459	1.141992
		.50	1.1516000*	.2703405	.012	.285875	2.017325
		1.00	.8100000	.2703405	.067	-.055725	1.675725

*At the level of the 0.05 threshold, there is a significant mean difference.
Intervention: Methanol-based extracts of *W. somnifera*
